# Supplementary material for: Ectopic expression of sericin enables efficient production of ancient silk with structural changes in silkworm
Source: Nat Commun. 2022 Oct 22;13:6295. doi: 10.1038/s41467-022-34128-5 (PMC9588020; doi:10.1038/s41467-022-34128-5)
Supplement: Supplementary file 1 — Supplementary Information [file 41467_2022_34128_MOESM1_ESM.pdf]

Supplementary Information for

Ectopic expression of sericin enables efficient production of ancient silk with structural changes in silkworm

Xuedong Chen[1,2,†], Yongfeng Wang[1,2,†], Yujun Wang[3,†], Qiuying Li[1,2], Xinyin Liang[1,2], Guang Wang[1,2], Jianglan Li[1,2], Ruji Peng[1,2], Yanghu Sima[1,2], Shiqing Xu[1,2 ✉].

[1]School of Biology and Basic Medical Sciences, Suzhou Medical College, Soochow University, Suzhou 215123, China; [2]National Engineering Laboratory for Modern Silk, Soochow University, Suzhou 215123, China; [3]Guangxi Key Laboratory of Beibu Gulf Marine Biodiversity Conservation, College of Marine Sciences, Beibu Gulf University, Qinzhou 535011, China. [†]These authors contributed equally: Xuedong Chen, Yongfeng Wang, Yujun Wang. [✉]To whom correspondence may be addressed. **Email**: szsqxu@suda.edu.cn.

Supplementary Information Text

1. Transgenic mutant silkworm screening and pure line establishment.

Construction of transgenic vector: Referring to the Dazo strain mRNA sequence (Gene ID: 100136948) published in the NCBI silkworm genome database (SilkDB, https://www.ncbi.nlm.nih.gov/genome/?term=Bombyx+mori), we cloned the 3120 bp sericin 3 protein (SER3) coding gene (*Ser*3) sequence specifically expressed in the middle silk gland from the N4W silkworm strain (Supplementary Sequence 1). The enhanced green fluorescent protein (EGFP) coding gene sequence (720 bp) was connected to *Ser*3, and the recombinant *Ser*3-*EGFP* nucleotide sequence was synthesized by BGI (Shenzhen, China). *SER3-EGFP* was spliced into the *Fib-H* 5' terminal sequence (2311 bp) (GenBank: AF226688.1, 61543–62437) and 3' terminal sequence (333 bp) (GenBank: AF226688.1, 79027–79359) to assemble the new gene *Ser*3' (6480 bp). *Ser*3' was inserted into the 3.5 kb plasmid pSLfa1180fa and further cloned into pBac[3xp3-DSredaf] via the AscI and FseI restriction sites to construct the recombinant vector pBac[3xP3-DsRedaf SV40-SER3'] for specific expression of recombinant *Ser*3 gene (*Ser*3') in the posterior silk gland in silkworms.

Preparation of silkworm eggs for transgenic injection: Parental eggs of N4W silkworms were incubated at 15 ℃ in a continuous dark environment until hatching. The larvae were fed fresh mulberry leaves, and the larvae, pupae and adults were protected at 25.0 ℃ ± 2.0 ℃ under natural light. After eclosion, the adult worms were mated for 6 h, and the eggs laid by the female moths were collected within 15 min. Microinjection was completed at 25 ℃ from 4 h to 8 h after oviposition. Before injection, silkworm eggs were soaked in ultra-pure water for 15 min to remove the sticky sundries, then soaked in 4% formaldehyde for 5 min for disinfection. After washing with water, they were quickly placed on slides under a dissecting microscope and dried for more than 30 min.

Transgenesis and mutant screening: The transgenic mutant line of Bombyx mori was constructed with *piggyBac* technology. According to a previously reported method[1], the auxiliary plasmid pHA3PIG was injected together with the constructed recombinant vector into silkworm eggs (Transfer-Man NK2 micromanipulator, FemtoJet 5247 micro syringe, Eppendorf). The concentration exceeded 500 ng/µL, and the injection volume was approximately 10 nL per egg. The injection site of eggs was 50%–75% from the

abdomen to the ovum, and the injection depth was 25%–35% the diameter of silkworm eggs. After injection, the silkworm eggs were quickly sealed with non-toxic glue (Topvalu) and incubated in a sterile environment of 25 °C and R.H. 90%.

The positive transgenic individuals were screened by observation of red fluorescence in the eyes of the 5th instar larvae and the green fluorescence in the cocoon (Supplementary Fig. 1b). A total of 85 adults were obtained from G0 generation larvae, of which 71 obtained offspring (G1 generation eggs), and the remaining 13 did not obtain offspring. Among the 71 G1 generation pedigrees, the number of positive pedigrees was 15, and the positive rate was 21% (Supplementary Fig. 1a). Individual selection was performed on 15 mutants of the S1 generation, and continuous mating was performed in the moth region (with close relatives of parents) to the G6 generation. The characteristics of red fluorescence of the eyes (Supplementary Fig. 1c) and green fluorescence of the cocoon (Supplementary Fig. 1d) were continuously used to screen until the G6 generation. After the detection of the mRNA (Supplementary Fig. 1e) and protein (Supplementary Fig. 1f) of the recombinant *Ser*3 gene in the posterior silk gland cells, and the detection of the insertion site of the recombinant foreign gene (Supplementary Fig. 1g), we confirmed the generation of a stable genetic single-copy transgenic silkworm system SER (*Ser*3'/ *Ser*3') (Supplementary Fig. 1b).

The insertion site of the piggyBac transposon in the SER mutant was detected by thermal asymmetric interleaving PCR (tail PCR). After comparison of the flanking sequence with the GenBank database sequence, the insertion site was located in chr.23 (scaf12: 4699379.. 4699384), and the insertion site was found not to be a functional gene sequence (Supplementary Fig. 1g). Therefore, the possibility of damage to functional gene sequence caused by random insertion of the piggyBac transposon in the genome was excluded.

2. Normal growth and improved production efficiency of cocoon silk in mutant SER silkworms.

The growth and development of silkworms and the production efficiency of the cocoon and silk were investigated. The developmental phenotype (Supplementary Fig. 2e) and body weight (Supplementary Fig. 2a) of SER larvae at the 5th instar showed no significant differences with respect to the wild-type (WT), and only the body weight and MRGR parameters at the beginning of the 4th instar were higher than those in the WT (Supplementary Fig. 2a-2c). No developmental phenotypic differences were observed in the silk glands of 5th instar larvae (Supplementary Fig. 2d), and no statistically significant difference was found in the

PSG/body parameter (Supplementary Fig. 2F), representing the development of an intact silk gland. However, the PSG/SG parameter representing the development of the posterior silk gland in the SER group (Supplementary Fig. 2e) was higher than that in the WT group, thus suggesting a potential advantage in the accumulation of silk material in the posterior silk gland of SER during the larval stage.

No significant difference was observed between the mutant and WT in the cocoon morphology and metamorphosis of the pupae produced by mature larvae, thus reflecting the comprehensive performance of growth and development and the health of the entire larval stage; no thin skin cocoon and semi-pupation phenomena, as commonly seen in transgenic silkworms, were observed (Supplementary Fig. 2g-2h). Moreover, we observed no significant difference in the cocoon weight (Supplementary Fig. 2i) or pupal weight (Supplementary Fig. 2j) between the mutant and WT. However, the cocoon layer weight of SER was 116.8% that of the WT group, from 0.104 g per cocoon in WT group to 0.123 g in the SER group (Supplementary Fig. 2k). The cocoon layer rate (cocoon silk production efficiency) of the SER silkworms was 114.8% higher than that of the the WT, from 10.64% in the WT group to 12.22% in the SER group (Supplementary Fig. 2l).

The effect of stress infection on larval survival was further investigated. All larvae died in the WT group at 78 h after *Escherichia coli* (*E. coli*) injection, whereas nearly 20% larvae in the SER group survived, and 16.7% mature larvae spun and cocooned (Supplementary Fig. 2k). At 24 h after infection with *Staphylococcus aureus* (*S. aureus*), all larvae in the WT group died, whereas 10% of the surviving larvae in the SER group successfully developed to spinneret cocoons (Supplementary Fig. 2l). The survival of SER larvae infected with bacteria under stress was higher than that of the WT.

3. Silk fibers produced by mutant SER have unique structure and properties.

Structure of Silk fibers: The cross-sections of silk fibers were observed by transmission electron microscopy (TEM). Sericin microsomes (SM) were found in the fibroin region of SER silk fibers, whereas SM were not present in WT silk fiber (Supplementary Fig. 3b). Vacuoles were seen in SM, thus reflecting the high concentration of sericin aqueous solution. We further confirmed that SER3 protein synthesized by the posterior silk glands of the mutant silkworms was successfully secreted into the fibroin of the silk fibers.

The infrared absorption spectrogram (wavelength range 4000–800 cm^{-1}) of degummed silk fibers was determined with an infrared spectrometer (Nicolet 5700, Thermo Electron Corporation, USA) with a resolution of 8 cm^{-1}. Each sample was scanned 256 times, and three samples were repeatedly analyzed. Spectral data were analyzed in OMNIC 9 software (Thermo Scientific) and PeakFit software (Seasolve, version 4.12). The amide I region deconvolution spectrum fitting method was used, and the peak position was determined by the second derivative peak position of the infrared spectrum, by using the same peak number (11 peaks) for curve fitting and secondary structure determination of FITR data as originally used in Supplementary Fig. 3e. The results showed that the β-sheet content was 47.69% in the WT group and 47.88% in the SER3 group (Supplementary Fig.3f). The difference between the two groups did not appear to be significant.

Silk fiber performance measurement: The obtained raw silk fibers were collected (one sample every 3 meters between 100 and 200 meters) to determine the mechanical properties or diameter of the silk. Meanwhile, ten samples were used to determine the diameter in the SER group and six samples were measured in the WT group. The diameter of the silk samples was measured with a digital microscope at 1000× magnification; multiple measurements were obtained from each sample, and the average diameter was calculated, and then the cross-sectional area was obtained ($S = \pi(d/2)^2$). The results indicated that the diameter and cross-sectional area of the mutant cocoon silk were smaller than those of the WT (Fig.S3g & S3h), and might further have affected the stress of silk fibers.

To test the application value of silk fibers produced by SER after improvements in the mechanical properties, we reeled ultra-fine raw silk of 9D-11D from cocoons, and wove ultra-dense (Supplementary Fig. 3c) and ultra-thin fabrics (Supplementary Fig. 3d), which could not be produced by the silk fiber from WT silkworms. These results demonstrated that the silk fiber produced by mutant silkworms has practical value for new engineering applications.

4. Review of technical strategy and protein synthesis efficiency of silk glands in transgenic silkworms.

Although the efforts to express and secrete exogenous proteins in the SGs of silkworms through transgenic technology to date have yielded many successful examples of genetic alterations, great challenges remain in greatly improving the expression efficiency of foreign proteins while maintaining the cocoon silk yield, especially the expression of high molecular weight proteins (~100kDa) in the posterior silk glands[2-8].

As shown in Supplementary Table 1, Bombyx mori expressed exogenous protein with a molecular weight greater than 100 kDa in the silk glands, and was prone to silk gland development deformities, decreased survival and significantly decreased cocoon silk production efficiency, thus resulting in thin layered cocoon shells[2-5]. Although abnormal cocoon silk yield has not been described in other reports, the expression of foreign proteins is generally not high. The highest content of foreign proteins reported is only 1.1% of the cocoon silk weight, and the expression in the posterior silk gland is less than 0.84% of the total cocoon silk[6-8]. The silk glands of silkworms are highly specialized tissues with self-silk protein expression, and the expression of foreign proteins must be improved.

5. SER silk fibers do not have significantly altered amino acid composition.

According to a previously described method[2], the amino acid composition of silk fiber was determined and quantified with an external standard method. The silk fiber samples containing sericin (raw silk) and textile silk fiber (degummed silk) with the outer sericin removed (0.2% $Na2CO_3$ boiled for 30 min and then dried) obtained from silk cocoon reeling were added to 8 mL 6.0 M HCl solution and filled with nitrogen. Hydrolysis was performed at 110 ± 1 °C for 24 h under airtight conditions. Then the samples were transferred to a volumetric bottle with 4.8 mL 10.0 mol/L NaOH with deionized water, and the volume was adjusted to 50 mL before filtration. The filtrate was centrifuged at 10000 r/min for 10 min, and 0.50 mL of supernatant was analyzed with high performance liquid chromatography (Ag1100, Agilent, Palo Alto, CA, USA).

The relative content of various amino acids was altered in raw silk. The relative content of serine and aspartic acid increased, whereas that of glycine, alanine and tyrosine decreased. Only the relative content of alanine in the degummed silk was higher than that in the WT. Other amino acids showed very little change (Supplementary Table 2), possibly because the mutant protein expressed in the posterior silk gland was secreted into the middle of the silk fibroin protein SER3, which has the same amino acid composition and similar relative content to that of the Fib-H/Fib-L/P25 protein polymer normally expressed by PSG (Supplementary Table 3).

6. SER silk fibroin has good biocompatibility comparable to that of classical silk fibroin.

The degummed silk fibers and non-absorbing polyester suture (NASS) were sterilized under high temperature and high pressure (121 °C, 30 min). L929 (ZQ0093, ZQXZ Biotech, Shanghai, China) mouse

fibroblasts were used for cytotoxicity testing. L929 cells were cultured overnight in 96 well plates with 100 µL Eagle's minimum essential medium (ZQ301, ZQXZ Biotech, Shanghai, China). The test fiber (1.0 mg /well) was soaked in the medium and gently cultured in the wells, and normal cultured L929 cells were used as a negative control. After continuous culture for 24 h and 48 h, a Live-Dead Kit (l3224L3224, Thermo Fisher Scientific, MA, USA) was used to distinguish living cells from dead cells, and an MTT Kit (C0009, Beyotime, Nantong, China) was used to detect cell proliferation. RAW264.7 cells (ZQ0098, ZQXZ Biotech, Shanghai, China) were used for cell inflammatory testing. The cells were cultured in 500 µL Dulbecco's modified Eagle medium (high glucose) (ZQ101, ZQXZ Biotech, Shanghai, China) in a 24 well plate overnight (the number of cells was as high as 3×10^{4}). The test fiber (10.0 mg/well) was soaked in the culture medium and gently cultured for 24 h and 48 h. The nitrous oxide content in the culture medium was determined with a Nitric Oxide Colorimetric Assay Kit (NO Kit) (S0021, Beyotime, Nantong, China).

Biocompatibility testing indicated that the fibroin fibers showed no adverse effects on the proliferation and growth of mammalian cells. Fibroin and L929 cells were co-cultured for 48 hours. The cell growth state (Supplementary Fig. 4a) and the proportion of dead cells (Supplementary Fig. 4b), as determined by Live-Dead staining, indicated that the fibroin fibers of SER were significantly better than the medical non-absorbable suture (NASS), and no statistical difference was observed relative to WT fibroin and the negative control (null). The MTT test results also indicated that the number of L929 cells in the SER group was significantly higher than that in the NASS group (Supplementary Fig. 4c). The content of the pro-inflammatory factor nitric oxide in the culture medium was significantly lower in the SER group than the NASS group (Supplementary Fig. 4d). SER silk fibroin had good biocompatibility comparable to that of the classical silk fibroin, although the sericin content was higher, and the structure of fibroin also changed.

Supplemental Figures and Tables

Supplementary Sequence 1 The nucleotide sequence of (5'→ 3')

```
AATAATACTG AAGGCCACGC TACCAGCAGC AGCTTCGATG AGCAAAGTTC TAGCGCTAGA     60
CAAAGCAGTA GCTCGTACCA GAGCCAGTCA TACAACAAAG ATTCTTCAAG TACCAATGAA    120
AGCAGTGATG GAGGCTCTGG TTCGGGCAGA ACCGGTTCAG CAGGAGAAAA TGGTGAGAAT    180
TCCGACGACA GCAGTGGTGC GACAAAAGGA AATAGCAGTA AATCTTCCAG CAGTTCTCAA    240
GGTCAAAGCG CAAGTAGTAG CAGCAGTGAT GAAAAATCAT CTCAGAGCAG CAGTAATAGT    300
AGCAACAACA GTAAATCAAG CAGCCAATCT TCCAGTAGCC AAAACAGTTC TGGTTCTAAG    360
GGCTCGGGAT CAGAAGAAAG CAGTAATGGA GGCTCTGGTT CGGGAAGAAC CGGTTCAGCG    420
GGAGGAACTG ATGAGGATTC CGACGACAGC AGTGGTGCGA CAAAAGGAAA TAGCAGTAAG    480
TCTTCCAGCA GTTCTCAAGG TCAAAGCGCA AGCAGTAGCA GCAGTGACGA AAACTCATCT    540
CAGAGCAGCA GTAATAGTAG CAACAACAGT AAATCAAGCA GCCAATCTTC CAGTGGCCAA    600
AACAGTTCTG GTTCTAAGGG CTCGGGATCC GAAGAAAGCA GTAATGGAGG CTCTGGTTCG    660
GGAAGAAACG GTTCAGTGGG AGGAACTGAT GAGGATTCCG ACGACAGCAG TGGTGCGACA    720
AAAGGAAATA GCAGTAAATC TTCCAGCAGC TCTCAAGGTC AAAGCGCAAG CAGTAGCAGC    780
AGTGATGAAA AATCATCTCA GAGCAGCAGT AATAGTAGCA ACAACAGTAA ATCAAGCAGC    840
CAATCTTCCA GTGGCCAAAA CAGTTCTGGT TCTAAGGGCT CGGGATCAGA AGAAAGCAGT    900
AATGGAGGCT CTGGTTCGGG AAGAAACGGT TCAGCGGGAG GAACTGATGA GGATTCCGAC    960
GACAGCAGTG GTGCGACAAA AGGAAATAGC AGTAAGTCTT CCAGCAGTTC TCAAGGTCAA   1020
AGCGCAAGCA GTAGCAGCAG TGATGAAAAA TCATCTCAGA GCAGCAGTAA TAGTAGCAAC   1080
AACAGTAAAT CAAGCAGCCA ATCTTCCAGT GGCCAAAACA GTTCTGGTTC TAAGGGCTCG   1140
GGATCAGAAG AAAGCAGTAA TGGAGGCTCT GGTTCGGGAA GAAACGGTTC AGCGGGAGGA   1200
ACTGATGAGG ATTCCGACGA CAGCAGTGGT GCGACAAAAG GAAATAGCAG TAAGTCTTCC   1260
AGCAGTTCTC AAGGTCAAAG CGCAAGCAGT AGCAGCAGTG ACGAAAAATC ATCTCAGAGC   1320
AGCAGTAATA GTAGCAACAA CAGTAAATCA AGCAGCCAAT CTTCCAGTGG CCAAAACAGT   1380
TCTGGTTCTA AGGGCTCGGG ATCAGAAGAA AGCAGTAATG GAGGCTCTGG TTCGGGAAGA   1440
ACCGGTTCAG CGGGAGAAAC TGATGAGGAT TCCGACGACA GCAGTGGTGC GACAAAAGGA   1500
AATAGCAGTA AGTCTTCCAG CAGTTCTCAA GGTAAAAGTG CAAGCAGTAG CAGCAGTGAC   1560
GAAAAATCAT CTCAGAGTAG CAGTAATAGT AGTAACAACA GTAAATCAAG CAGTCAATCT   1620
TCGAGTAGCA ACAATAGTTC TGGTTCTAAG GGCTCGGGAT CAGAAGAAAG CAGTAATGGA   1680
GGCTCTGGTT CGGGAAGAAC CGGTTCAGCG GGAGGAAGTG ATGAGGATTC CGACGACAGC   1740
AGTGGTGCGA CAAAAGGAAA TAGCAGTAAG TCTTCCAGCA GCTCTCAAGG TCAAAGCGCA   1800
AGCAGTAGCA GCAGTGACGA AAAATCATCA CAGAGCAACA GTAATAGTAG CAATAACAGT   1860
AAATCAAGTA GCCAATCTTC GAGTAGCAAC AACAGTTCTG GTTCTAAGGG CTCGGGATCA   1920
GAAGAAAGCA GTAATGGAGG CTCTGGTTCG GGAAGAACCG GTTCAGCGGG AGGAACTGAT   1980
GAGGATTCCG ATGACAGCAG TGGTGCGACA AAAGGAAATA GCAGTAAGTC TTCCAGCAGT   2040
TCTCAAGGTA AAAGCGCAAG CAGTAGCAGC AGTGACGAAA AATCATCTCA GAGCAGCAGT   2100
AATAGTAGTA ATAACAGTAA ATCAAGCAGC CAATCGTCCA GTAGCAAGAA CAGTTCTGGT   2160
TCTAAGGGCT CGGGATCAGA AGAAAGCAGT AATGGAGGCT CTGGTTCGGG AAGAACCGGT   2220
TCAGCGGGAG GAACTGATGA GGATTCCGAC GACAGCAGTG GTGCGACAAA AGGAAATAGC   2280
AGTAAGTCTT CCAGCAGTTC TCAAGGTAAA AGCGCAAGCA GTAGCAGCAG TGACGAAAAA   2340
TCATCTCAGA GTAACAGTAA TAGTAGTAAC AACAGTAAAT CAAGCAGTCA ATCTTCGAGT   2400
AGTAAGAACA GTTCTGGTTC TAAGGGCTCG GGATCAGAAG AAAGCAGTAA TGGAGGCTCT   2460
GGTTCGGGAA GAACCGGTTC AGCGGGAGGA ACTGATGAGG ATTCCGACGA CAGCAGTGGT   2520
GCGACAAAAG GAAATAGCAG TAAGTCTTCC AGCAGTTCTC AAGGTAAAAG CGCAAGCAGT   2580
AGCAGCAGTA ACGAAAAATC ATCTCAGAGT AGCAGTAATA GTAGTAACAA CAGTAAATCA   2640
AGCAGTCAAT CTTCGAGTAG CAAGAACAGT TCTGGTTCTA AGGGCTCTGG ATCAAGTGAA   2700
AGTGGTGATA AAAAGTCCAG TTCTCGAGGA AGTTCTGGTG ACAACTCAGA CGATGACCAA   2760
ACTGATTCAG CCAGATCAAA TAGTAAGCGT TCCACAAGCT CTGATGCGTC CACTAAAAAA   2820
AGTTCGTCTA GAAAGAGCTC CAACCACCGT AGTAGCAGAA GTCAGCAAGC TCATAGTAGC   2880
AGCAGTAAAC AAGCCCAAAG CAGCAGTAGT CAACAAGCCC AAAATAGCAG AAGTCAGCAA   2940
GCTCATAGTA GCAGAAGTCA GCAAGCTCAT AGTAGCAGCA GTAAACAAGC CCAAAGCAGC   3000
AGTAGTAAAC AAGCCCAAAG TAGCAGCAGT AAACAAGCCC AAAGCAGCAG TAGTAAACAA   3060
GCCCAAAGTA GCAGTAGTCA ACAAGCCCAA AGTAGCAGAA GTCAGCAAGC TCATAGTAGC   3120
```

a

Target gene	Injected eggs	Hatched (%)	G0 adults	G1 broods	Positive broods	Mutant (%)
BmSer3	1820	32	85	71	15	21

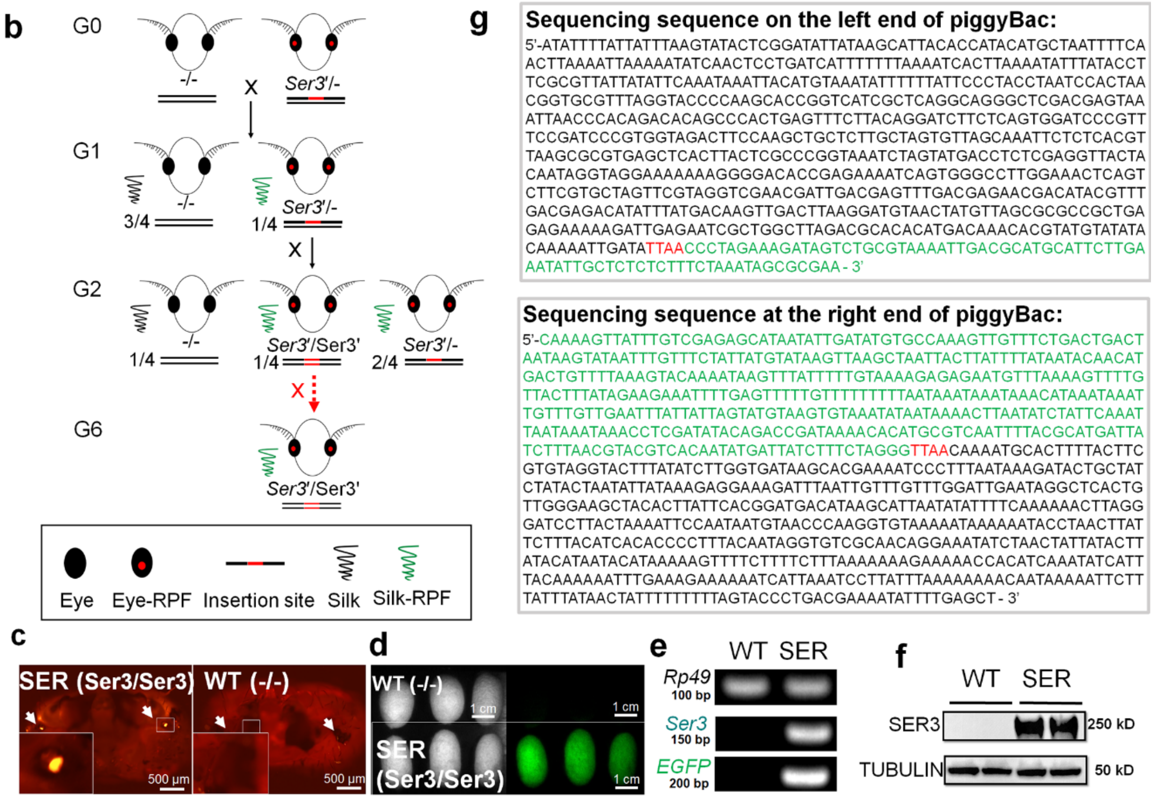

Supplementary Fig 1. Establishment of mutant silkworm lines expressing the SER3 gene in posterior silk glands. **(a)** Mutation rate of transgenic injected eggs. **(b)** Homozygous mutant screening strategy. G0 generation heterozygote mated with WT to produce the heterozygote G1 generation, which began to self-cross in the moth region. Homozygous mutants were screened after the G2 generation. **(c-f)** Mutant identification. WT, wild-type; SER (*Ser*3'/*Ser*3'), homozygous mutant. (**c**) Transgenic mutants expressed red fluorescent protein RFP in the eyes at the 3rd day of 5th instar larvae. **(d)** The cocoon silk of transgenic mutant showed green fluorescence. **(e)** *Ser3* gene mRNA expressed in the posterior silk gland cells, detected by RT-PCR. **(f)** SER3 protein expressed by posterior silk gland cells, detected by western blotting**.** EGFP localization showed that the recombinant SER3 protein had two types: single molecule and dimer, of which dimer was the main type. The recombinant SER3 in Supplementary Fig.1f is a dimer type. **(g)** Tail-PCR sequencing analysis of the insertion site of the recombinant SER3 gene**.** The *piggyBac* tag "TTAA" is marked in red, and the *piggyBac* arm is marked in green. n=3 samples. Each tissue sample was collected from three female individuals, and each sample was measured three times. Image data are representative of three independent experiments unless otherwise stated.

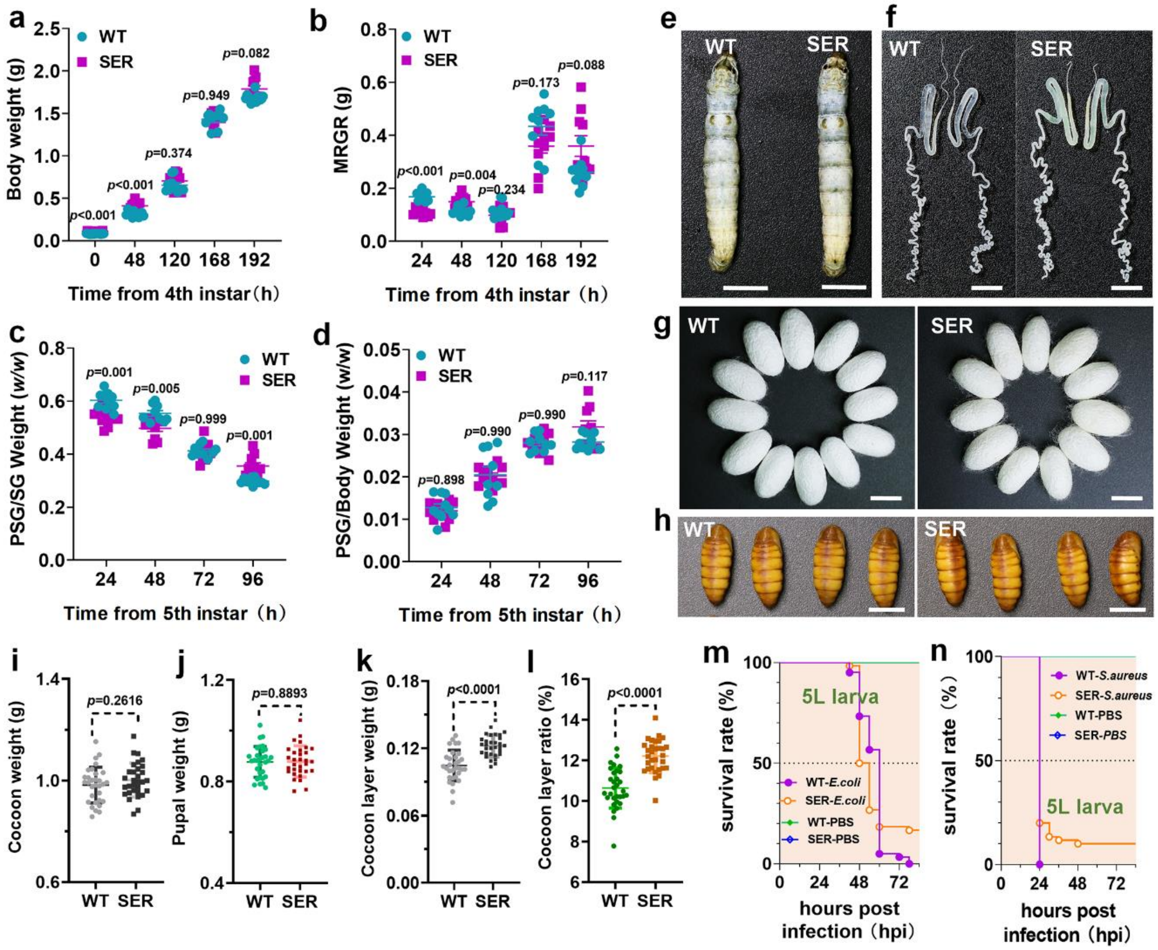

Supplementary Fig 2. The mutant SER silkworm growth and cocoon silk production efficiency. (**a**) Body weight. The 4th instar larvae were given mulberry for the first time (0 h), and ten larvae of the same sex (male) were randomly selected and weighed individually every 24 h. (**b**) The mean relative growth rate (MRGR) within 24 h was further calculated on the basis of the body weight. (**c&d**) Development of the posterior silk gland. After the 5th instar larvae were given mulberry for the first time (0 h), ten larvae of the same sex (male) were randomly selected every 24 h. Complete silk glands were dissected and weighed to calculate the ratio of posterior silk gland weight to silk gland weight (PSG/SG) (**c**), and the ratio of posterior silk gland weight to body weight (PSG/body) (**d**). (**e**) 5L larvae. (**f**) 5L3d larval silk gland. **(g)** Cocoon. **(h)** Silkworm pupa. **(i)** Cocoon weight. (**j**) Pupal weight. **(k)** Cocoon layer weight. (**l**) Cocoon layer ratio. At 72 h after cocooning, 31 cocoons of the female were randomly selected and weighed to calculate the percentage of cocoon shell weight in the cocoon weight (cocoon layer ratio). **(m) & (n)** Survival rate after infection. Female larvae at 5L2d with similar body weights (1.00G ± 0.10 g) were injected with 10 μL bacterial solution from the second abdominal ganglion membrane with a glass capillary (n=60 larvae). The OD_{600} value of the bacterial solution was 1.800 for *Escherichia coli (E. coli)* and 0.165 for *Staphylococcus aureus* (*S. aureus*). The control treatment was PBS. The number of surviving larvae was counted every 6 h until the larvae formed cocoons or all individuals died. n=10 individual larva in (**a-d**), n=31 individual larva in (**i-l**). Data were presented as mean ± SEM. For (**a-d**), Holm-Sidak t-test analysis was used and the p value obtained was the adjusted p value. For (**i-l**), the unpaired t test analysis was used. "$p \leq 0.05$" indicates a significant difference between the two groups, and "$p > 0.05$" indicates no significant difference between the two groups. Bar=1cm in (**e-h**).

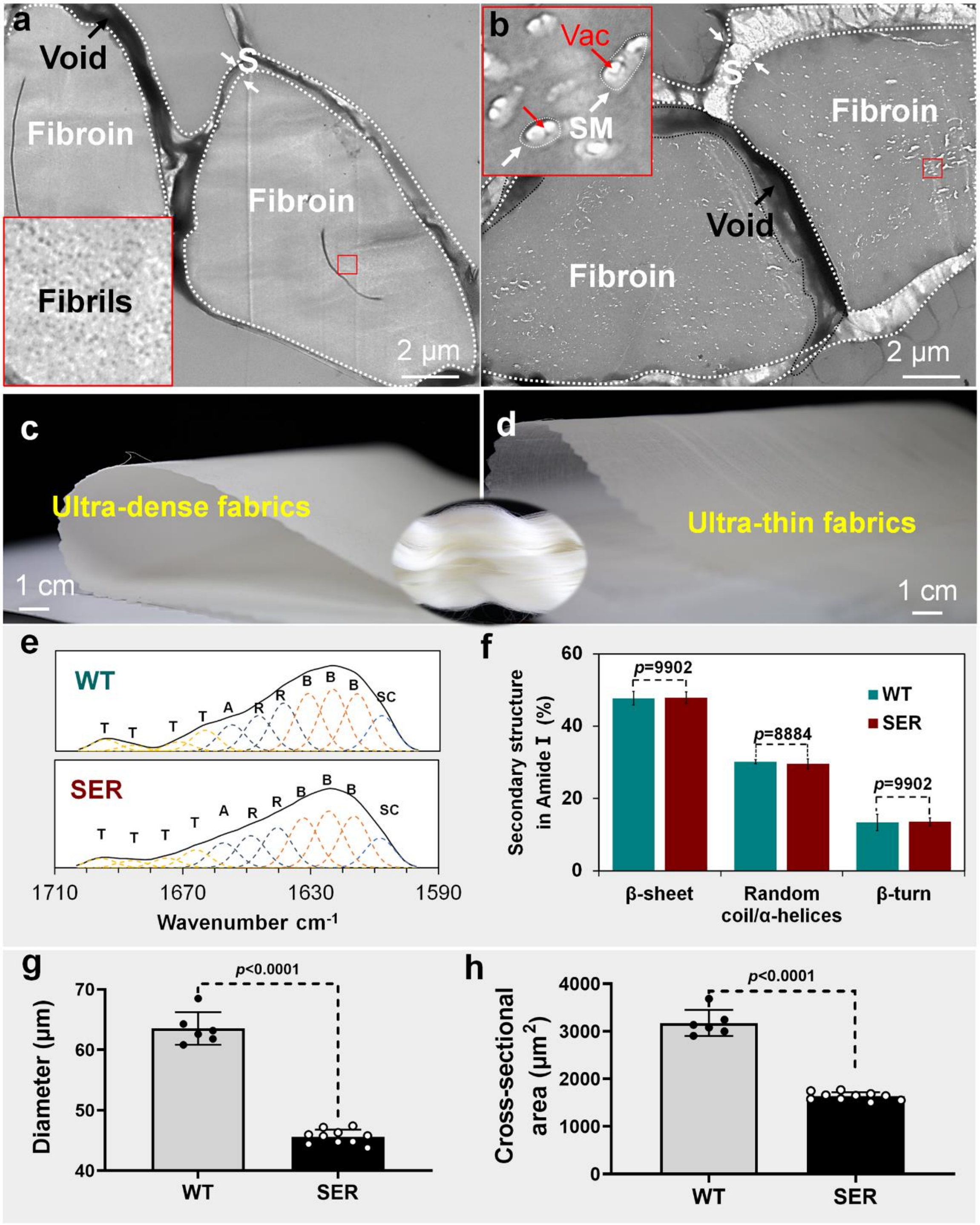

Supplementary Fig 3. TEM images of silk fibers and special silk fabrics produced by the mutant silkworms. Cross-sections of silk fibers of (**a**) wild-type (WT) and (**b**) mutant (SER). SER3 proteins expressed in the posterior silk glands were found to be dispersed in cocoon fibers. Fibroin, the cocoon silk core composed of silk fibroin protein; S, the outer layer of cocoon silk composed of sericin protein; SM, sericin microsome, in the silk core. Vac, vacuoles. **(c)** Ultra dense fabrics**. (d)** Ultra-thin fabrics. These silk fabrics showed advantages in the mechanical properties of SER fibers; in contrast, the mechanical properties

of WT fibers cannot enable the production of such products. (**e**) Deconvolution of amide I bands in silk fibers, analyzed by FITR. The amide I band (1700–1600 cm^{-1}) was deconvoluted with the Fourier self-deconvolution method to determine the changes in silk fiber β-sheets, random coils and α-helices. The black solid line is the amide I band spectrum, and the dotted line is a separate deconvolution peak. Peak abbreviation mark: T, β-turn; A, α-helices; R, random coil, B, β-sheets, SC, side chain. (**f**) Statistics of protein secondary structure components in silk fibers (n=3). **(g)** Diameter & **(h)** the cross-sectional area of the raw silk. 10 samples were measured to determine the diameter in SER group and 6 samples were measured in WT group. Data were presented as mean ± SEM. "$p \leq 0.05$" indicates a significant difference between the two groups, and "$p >$ 0.05" indicates no significant difference between the two groups.

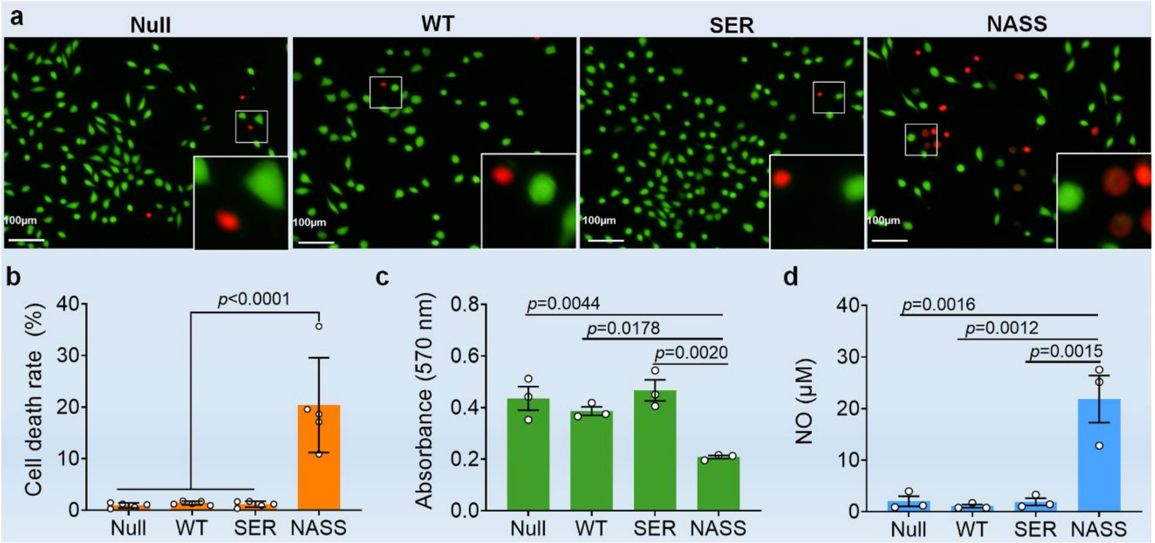

Supplementary Figure 4. The mutant SER silk has good biocompatibility. (a-d) Cytotoxicity and inflammation testing. Fibroin mixed culture cells for 48 h. **(a)** Cell morphology, assessed by Live-Dead staining and **(b)** the proportion of dead cells. **(c)** Relative proliferation rate of L929 cells, detected with the MTT method. **(d)** Content of nitric oxide in the medium of RAW264.7 cells. Null, control. WT, fibroin of WT. SER, fibroin of SER; NASS, medical non-absorbable suture. n=3 biologically independent cells. Data were presented as mean ± SEM. Ordinary one-way ANOVA (Tukey's multiple comparisons test). "$p \leq 0.05$" indicates a significant difference between the two groups, and "$p > 0.05$" indicates no significant difference between the two groups. Image data are representative of three independent experiments unless otherwise stated.

Supplementary Table 1. Recombinant protein expression levels in silk glands of transgenic silkworms.

Year	Author	Exogenous protein (molecular weight)	Silkworm strain	Promoter	Expression level of products
**2021**	Tang *et al.*[9]	Pyriform spidroin 1 (~47 kDa)<br>Aggregate spider glue 1 (~43 kDa)	Lan 10	Fib-H	blank
**2020**	Minagawa *et al.*[3]	Fibrinogen (~340 kDa)	w1-pnd	Ser1	approx 2% (w/w) of cocoon shell weight, thin-layered cocoon shells
**2020**	Leem *et al.*[10]	Photoelectric protein (~25 kDa)	Keumokjam	Fib-H	approx 12.6% (w/w) of cocoon shell weight
**2019**	Zhang *et al.*[11]	Spider silk protein (120 kDa, 300 kDa)	Haoyue	Fib-H & Fib-L	blank
**2019**	Zhang *et al.*[12]	Recombinant human vascular endothelial growth factor (~23 kDa)	Nistari	Ser1	blank
**2018**	Xu *et al.*[13]	Major ampullate spidroin-1 (~67 kDa)	Nistari	Fib-H	approx 35.2% (w/w) of cocoon shell weight, thin-layered cocoon shells
**2018**	Qian *et al.*[14]	Human serum albumin (66 kDa)	Lan10	Fib-L	approx 1.74% (w/w) of cocoon shell weight
**2018**	Sachi *et al.*[15]	Feline interferon (18.9 kDa)	w1-pnd	Ser1	approx 0.17% (w/w) of cocoon shell weight
**2018**	Chen *et al.*[16]	Human platelet-derived growth factor (~27 kDa)	Dazao	Ser1	approx 0.024% (w/w) of cocoon shell weight
**2018**	You *et al.*[17]	Major ampullate spidroin (39.2 kDa, 133.5 kDa, 171.2 kDa)	Lan10	Fib-H	approx 1.67%-3.09% (w/w) of Fib-L
**2017**	Otsuki *et al.*[2]	Butterfly cytotoxin Pierisin-1A (98 kDa)	w1-pnd	Fib-H	thin-layered cocoon shells
**2017**	You *et al.*[18]	Major royal jelly protein-1 (55 kDa)	Lan10	Fib-L	approx 0.78% (w/w) of cocoon shell weight
**2017**	Goo *et al.*[19]	Cyan fluorescent protein (27 kDa)	Kumokjam	Fib-H	blank
**2016**	Wang *et al.*[20]	Ca-binding (12.1 kDa)	Lan10	Fib-L	blank
**2015**	Tada *et al.*[21]	Anti-CD20 monoclonal antibody (150 kDa)	w1-pnd	Ser1	250 μg per cocoon
**2015**	Li *et al.*[22]	Cecropin (68.7 kDa)	GaoBai	Fib-L	blank
**2015**	Wang *et al.*[4]	HPL (~120 kDa)	N4	Fib-H	approx 18.85% (w/w) of fibroin,<br>thin-layered cocoon shells
**2015**	Wang *et al.*[23]	Human acidic fibroblast growth factor (15.8 kDa)	Dazao	Ser1	pprox 3.6%-6.7% (w/w) of cocoon shell weight
**2014**	Wang *et al.*[24]	Human acidic fibroblast growth factor (15.8 kDa)	Dazao	Ser1	approx 0.07% (w/w) of cocoon shell weight
**2014**	Kuwana *et al.*[6]	Spider dragline silk (~100 kDa)	C515	Fib-H	0.37 to 0.61% (w/w) native silkworm fibroin
**2014**	Sato *et al.*[25]	Single-chain variable fragment (~26 kDa)	pnd-w1	Fib-L	5%-10% of the total Fib-L

**2014**	Song *et al.*[26]	Human insulin-like growth factor (7.5 kDa)	Dazao×Haoyue	Ser1	approx 162.7 ng/g of cocoon shell weight
**2014**	Li *et al.*[27]	Antibacterial silk (~4 kDa)	GaoBai	Fib-L	blank
**2013**	Wang *et al.*[28]	DsRed (25.9 kDa)	Dazao	Ser1	9.5 % (w/w) of cocoon shell weight
**2013**	Iizuka *et al.*[29]	EGFP, DsRed & Monomeric Kusabira orange (26.9 kDa, 25.9 kDa, 24.7 kDa)	Pnd-w1	Fib-H	blank
**2012**	Xue *et al.*[30]	Human granucyto-macrophage (22 kDa)	GaoBai	Fib-H	approx 2.70 ng/g of freeze dried powdered posterior silk gland
**2012**	Teulé *et al.*[5]	Partial spider silk fibroin sequence (106 kDa)	pnd-w1	Fib-H	approx 2%-5% (w/w) of cocoon shell weight,thin-layered cocoon shells
**2011**	Li Y *et al.*[31]	Human insulin-like growth factor (7.5 kDa)	GaoBai	Fhx	approx 150 ng/g of fresh posterior silk gland
**2010**	Zhao *et al.*[32]	EGFP (26.9 kDa)	N4	Fib-H	15% (w/w) of silk fibroin
**2010**	Adachi *et al.*[33]	Non-triple helical collagen α chain (~90 kDa)	Pnd-w1	Ser1	8% of the dried cocoon silk
**2010**	Wen *et al.*[34]	Spider dragline silk (~67 kDa)	N4/Pnd-w2	Ser1	1.2 times the intensity of silk compared to control
**2010**	Tatematsu *et al.*[35]	EGFP (26.9 kDa)	Pnd-w1-Nd -Sd Pnd-w1	Ser1	500 µg per larva
**2010**	Zhu *et al.*[36]	Partial spider silk fibroin sequence (~67 kDa)	Pnd-w1	Fib-H	5% (w/w) of silk fibroin
**2009**	Iizuka *et al.*[7]	Mouse monoclonal antibody (~200 kDa)	Pnd-w1	Ser1	1.1% of the dried cocoon silk
**2009**	Tateno *et al.*[37]	Human µ-opioid receptor (~44 kDa)	Pnd-w1	Fib-L	150-250 ng per 1g silk gland
**2009**	Zhao *et al.*[38]	Human insulin-like growth factor-I (~22 kDa)	Haoyue	Ser1	2.44 ng/g of middle silk gland
**2007**	Kurihara *et al.*[39]	Feline interferon (30.9 kDa, 40.9 kDa)	Pnd-w1	Fib-H	6% of the dried cocoon silk
**2007**	Ogawa *et al.*[40]	Human serum albumin (66.5 kDa)	Pnd-w1	Ser1	0.3% of the dried cocoon silk
**2007**	Yanagisawa *et al.*[41]	Collagen & RGD (27.72 kDa, 6.65 kDa)	Pnd-w1 & Nd-s^D	Fib-L	0.2% & 0.6% of the dried cocoon silk
**2006**	Hino *et al.*[42]	Human basic fibroblast growth factor (~18 kDa)	Pnd-w1	Fib-L	0.04% of the fibroin
**2005**	Inoue *et al.*[43]	Fib-L-GFP (25.9 kDa)	C108 & Nd-s^D	Fib-L	10% of the dried cocoon silk
**2003**	Tomita *et al.*[8]	Human type III procollagen (138 kDa)	Pnd-w1	Fib-L	0.84% of the dried cocoon silk

Supplementary Table 2. Percentages of amino acids in silk fibers from SER and WT.

Amino acid (%)	Raw silk		Degummed silk	
	WT	SER	WT	SER
Glycine	30.45 ± 0.51	27.70 ± 0.71	39.56 ± 0.74	39.46 ± 0.09
Alanine	22.75 ± 0.40	20.44 ± 0.52	29.9 ± 0.14	30.41 ± 0.08
Serine	12.86 ± 0.27	13.21 ± 0.52	9.25 ± 0.06	9.22 ± 0.01
Tyrosine	7.18 ± 0.20	6.41 ± 0.37	8.40 ± 0.28	8.75 ± 0.26
Aspartic acid	5.90 ± 0.12	6.75 ± 0.46	1.98 ± 0.15	1.79 ± 0.01
Glutamic acid	3.33 ± 0.10	3.63 ± 0.22	1.95 ± 0.03	1.84 ± 0.03
Valine	3.15 ± 0.06	3.12 ± 0.09	2.81 ± 0.10	2.89 ± 0.02
Threonine	2.73 ± 0.03	3.01 ± 0.18	0.90 ± 0.03	0.91 ± 0.01
Arginine	1.85 ± 0.01	2.13 ± 0.14	0.75 ± 0.01	0.69 ± 0.00
Lysine	1.27 ± 0.01	1.52 ± 0.13	0.31 ± 0.01	0.31 ± 0.01
Phenylalanine	1.18 ± 0.02	1.15 ± 0.03	1.17 ± 0.03	1.16 ± 0.00
Isoleucine	0.94 ± 0.05	1.00 ± 0.05	0.77 ± 0.03	0.71 ± 0.01
Leucine	0.88 ± 0.04	0.97 ± 0.05	0.63 ± 0.02	0.54 ± 0.00
Proline	0.71 ± 0.12	0.74 ± 0.02	0.66 ± 0.06	0.57 ± 0.07
Histidine	0.73 ± 0.08	0.77 ± 0.04	0.36 ± 0.02	0.35 ± 0.01
Methionine	0.25 ± 0.01	0.28 ± 0.02	0.07 ± 0.06	0.05 ± 0.01
Cysteine	0.11 ± 0.09	0.22 ± 0.03	0.02 ± 0.01	0.06 ± 0.07

Supplementary Table 3. Percentages of amino acid residues in Fib-H, Fib-L, P25 and recombinant SER3' proteins.

Amino acid (%)	Fib-H	Fib-L	P25	SER3'
Alanine	30.30	14.10	7.30	4.70
Arginine	0.30	3.80	5.90	2.50
Asparagine	0.40	6.90	5.90	6.90
Aspartic acid	0.50	6.50	6.40	5.90
Cysteine	0.10	1.10	4.10	0.50
Glutamine	0.20	5.70	3.60	5.90
Glutamic	0.60	2.30	3.20	4.60
Glycine	45.90	8.40	4.10	11.80
Histidine	0.10	1.90	3.60	1.10
Isoleucine	0.20	8.00	6.40	1.50
Leucine	0.10	7.60	10.00	1.90
Lysine	0.20	1.50	3.20	6.70
Methionine	0.10	0.80	0.90	0.70
Phenylalanine	0.60	3.10	6.80	1.30
Proline	0.30	3.40	5.50	0.90
Serine	12.10	9.50	6.40	34.80
Threonine	0.90	3.10	5.50	4.20
Tryptophan	0.20	0.80	1.40	0.10
Tyrosine	5.30	4.20	4.50	1.70
Valine	1.80	7.30	5.50	2.50

Supplementary Table 4. SqRT-PCR or qPCR primer sequence

Gene name	Gene ID	Primers	Sequences（5' → 3'）
Rp49	778453	*Rp49-S*	CAGGCGGTTCAAGGGTCAATAC
		Rp49-A	TACGGAATCCATTTGGGAGCAT
Ser3	100136948	*Ser3-S*	CGCTGCGGGTACAGGTAT
		Ser3-A	GCACTTCCACCGACGAGA
EGFP	20473140	*EGFP-S*	CACCTACGGCAAGCTGACCC
		EGFP-A	CGCTCCTGGACGTAGCCTTC
Fib-H	693030	*Fib-H-S*	ACAAGGTGCAGGAAGTGC
		Fib-H-A	AGCAATTCACACAAGGCAGT
Fib-L	693047	*Fib-L-S*	CCGGAGGTGGAAGAATCTAT
		Fib-L-A	GGTTATGTAGGCAGCGATGT
P25	100146105	*P25-S*	CCCTGCTACTTGGACGATT
		P25-A	GATTATGGTCGACGTAGGTG

References

1. Wang, Y. & Nakagaki, M. Editing of the heavy chain gene of *Bombyx mori* using transcription activator like effector nucleases. *Biochem Biophys Res Commun* **450**, 184-188 (2014).
2. Otsuki. R. et al., Bioengineered silkworms with butterfly cytotoxin-modified silk glands produce sericin cocoons with a utility for a new biomaterial. Proc. Natl. Acad. Sci. USA **114**, 6740-6745 (2017).
3. Minagawa, S. et al. Production of a correctly assembled fibrinogen using transgenic silkworms. Transgenic Res **29**, 339-353 (2020).
4. Wang, H. et al. High yield exogenous protein HPL production in the Bombyx mori silk gland provides novel insight into recombinant expression systems. *Sci Rep* **5**, 13839 (2015).
5. Teulé, F. et al. Silkworms transformed with chimeric silkworm/spider silk genes spin composite silk fibers with improved mechanical properties. Proc. Natl. Acad. Sci. USA **109**, 923-928 (2012)

6. Kuwana, Y. et al. High-toughness silk produced by a transgenic silkworm expressing spider (Araneus ventricosus) dragline silk protein. PLoS One **9**, e105325 (2014)
7. Iizuka, M. et al. Production of a recombinant mouse monoclonal antibody in transgenic silkworm cocoons. FEBS. J 276, 5806-5820 (2009)
8. Tomita, M. et al. Transgenic silkworms produce recombinant human type III procollagen in cocoons. Nat. Biotechnol 21, 52-56 (2003).
9. Tang, X. et al. High mechanical property silk produced by transgenic silkworms expressing the spidroins PySp1 and ASG1. *Sci Rep* **11**, 20980 (2021).
10. Leem, J. W. et al. Photoelectric Silk via Genetic Encoding and Bioassisted Plasmonics. *Adv. Biosyst* **4**, e2000040 (2020).
11. Zhang, X. et al. CRISPR/Cas9 Initiated Transgenic Silkworms as a Natural Spinner of Spider Silk. *Biomacromolecules* **20**, 2252-2264 (2019).
12. Zhang, T. et al. Expression and characterization of recombinant human VEGF165 in the middle silk gland of transgenic silkworms. *Transgenic Research* **28**, 601–609 (2019).
13. Xu, J. et al. Mass spider silk production through targeted gene replacement in *Bombyx mori*. *Proc. Natl. Acad. Sci. USA* **115**, 8757-8762 (2018).
14. Qian, Q. et al. High-efficiency production of human serum albumin in the posterior silk glands of transgenic silkworms, *Bombyx mori L*. *PLoS One* **13**, e0191507 (2018).
15. Sachi M, Yuzuru N, Masahiro T, et al. Novel recombinant feline interferon carrying N-glycans with reduced allergy risk produced by a transgenic silkworm system. *BMC Veterinary Research* 14, 260 (2018).
16. Chen, W. J. et al. Transgenic Silkworm-Based Silk Gland Bioreactor for Large Scale Production of Bioactive Human Platelet-Derived Growth Factor (PDGF-BB) in Silk Cocoons. *Int. J. Mol. Sci* **19**, 2533 (2018).
17. You, Z. et al. Extraordinary Mechanical Properties of Composite Silk Through Hereditable Transgenic Silkworm Expressing Recombinant Major Ampullate Spidroin. *Sci Rep* **8**, 15956 (2018).
18. You, Z. et al. Transgenic silkworms secrete the recombinant glycosylated MRJP1 protein of Chinese honeybee, *Apis cerana cerana*. *Transgenic. Res* **26**, 653-663 (2017).

19. Goo, T. W. et al. Expression of the cyan fluorescent protein in fibroin h-chain of transgenic silkworm. *Int. J. Ind. Entomol* **34**, 11-15 (2017).
20. Wang, S. et al. Characterization of Transgenic Silkworm Yielded Biomaterials with Calcium-Binding Activity. *Plos. One* **11**, e0159111 (2016).
21. Tada. M. et al. Characterization of anti-CD20 monoclonal antibody produced by transgenic silkworms (*Bombyx mori*). *MAbs* **7**, 1138-1150 (2015).
22. Li, Z. et al. Construction of transgenic silkworm spinning antibacterial silk with fluorescence. *Mol. Biol. Rep* **42**, 19-25 (2015).
23. Wang, F. et al. Large-scale production of bioactive recombinant human acidic fibroblast growth factor in transgenic silkworm cocoons. *Sci. Rep* **5**, 16323 (2015)
24. Wang, F. et al. Advanced silk material spun by a transgenic silkworm promotes cell proliferation for biomedical application. *Acta Biomaterialia*. **10**, 4947-4955 (2014).
25. Sato, M. et al. Production of scFv-conjugated affinity silk film and its application to a novel enzyme-linked immunosorbent assay. *Sci. Rep* **4**, 4080 (2014).
26. Song, Z. et al. Reducing blood glucose levels in TIDM mice with an orally administered extract of sericin from hIGF-I-transgenic silkworm cocoons. *Food. Chem. Toxicol* **67**, 249-254 (2014).
27. Li, Z. et al. Construction of transgenic silkworm spinning antibacterial silk with fluorescence. *Mol. Biol. Rep* **42**, 19-25 (2014).
28. Wang, F. et al. An optimized sericin-1 expression system for mass-producing recombinant proteins in the middle silk glands of transgenic silkworms. *Transgenic. Res* **22**, 925-938 (2013).
29. Iizuka, T. et al. Colored fluorescent silk made by transgenic silkworms. *Adv. Funct. Mater* **23**, 5232-5239 (2013).
30. Xue, R. et al. Expression of hGM-CSF in silk glands of transgenic silkworms using gene targeting vector. *Transgenic. Res* **21**, 101-111 (2012).
31. Li, Y. et al. Expression of the hIGF-I gene driven by the Fhx/P25 promoter in the silk glands of germline silkworm and transformed BmN cells. *Biotechnol. Lett* **33**, 489-494 (2011).
32. Zhao, A. et al. New and highly efficient expression systems for expressing selectively foreign protein in the silk glands of transgenic silkworm. *Transgenic. Res* **19**, 29-44 (2010).

33. Adachi, T. et al. Production of a non-triple helical collagen alpha chain in transgenic silkworms and its evaluation as a gelatin substitute for cell culture. *Biotechnol. Bioeng* **106**, 860-870 (2010).

34. Wen, H. et al. Transgenic silkworms (*Bombyx mori*) produce recombinant spider dragline silk in cocoons. *Mol. Biol. Rep* **37**, 1815-1821 (2010).

35. Tatematsu, K. et al. Construction of a binary transgenic gene expression system for recombinant protein production in the middle silk gland of the silkworm *Bombyx mori*. *Transgenic. Res* **19**, 473-487 (2010).

36. Zhu, Z. et al. Mechanical properties of regenerated *Bombyx mori* silk fibers and recombinant silk fibers produced by transgenic silkworms. *J Biomater Sci Polym Ed* **21**, 395-411 (2010).

37. Tateno, M. et al. Production and characterization of the recombinant human mu-opioid receptor from transgenic silkworms. *J. Biochem* **145**, 37-42 (2009).

38. Zhao, Y. et al. Expression of hIGF-I in the silk glands of transgenic silkworms and in transformed silkworm cells. *Sci. China. C. Life. Sci* **52**, 1131-1139 (2009).

39. Kurihara, H., Sezutsu, H., Tamura, T. & Yamada, K. Production of an active feline interferon in the cocoon of transgenic silkworms using the fibroin H-chain expression system. *Biochem. Biophys. Res. Commun* **355**, 976-980 (2007).

40. Ogawa, S., Tomita, M., Shimizu, K. & Yoshizato, K. Generation of a transgenic silkworm that secretes recombinant proteins in the sericin layer of cocoon: production of recombinant human serum albumin. *J. Biotechnol* **128**, 531-544 (2007).

41. Yanagisawa, S. et al. Improving cell-adhesive properties of recombinant Bombyx mori silk by incorporation of collagen or fibronectin derived peptides produced by transgenic silkworms. *Biomacromolecules* **8**, 3487-3492 (2007).

42. Hino, R., Tomita, M. & Yoshizato, K. The generation of germline transgenic silkworms for the production of biologically active recombinant fusion proteins of fibroin and human basic fibroblast growth factor. *Biomaterials* **27**, 5715-5724 (2006).

43. Inoue, S. et al. A fibroin secretion-deficient silkworm mutant, Nd-sD, provides an efficient system for producing recombinant proteins. *Insect. Biochem. Mol. Biol* **35**, 51-59 (2005).
