# Supplementary material for: Ectopic expression of sericin enables efficient production of ancient silk with structural changes in silkworm
Source: Nat Commun. 2022 Oct 22;13:6295. doi: 10.1038/s41467-022-34128-5 (PMC9588020; doi:10.1038/s41467-022-34128-5)
Supplement: Supplementary file 3 — Reporting Summary [file 41467_2022_34128_MOESM3_ESM.pdf]

nature portfolio

Corresponding author(s): Shiqing Xu

Last updated by author(s): Sep 22, 2022

Reporting Summary

Nature Portfolio wishes to improve the reproducibility of the work that we publish. This form provides structure for consistency and transparency in reporting. For further information on Nature Portfolio policies, see our Editorial Policies and the Editorial Policy Checklist.

Statistics

For all statistical analyses, confirm that the following items are present in the figure legend, table legend, main text, or Methods section.

n/a	Confirmed	
☐	☒	The exact sample size (n) for each experimental group/condition, given as a discrete number and unit of measurement
☐	☒	A statement on whether measurements were taken from distinct samples or whether the same sample was measured repeatedly
☐	☒	The statistical test(s) used AND whether they are one- or two-sided<br>*Only common tests should be described solely by name; describe more complex techniques in the Methods section.*
☒	☐	A description of all covariates tested
☐	☒	A description of any assumptions or corrections, such as tests of normality and adjustment for multiple comparisons
☐	☒	A full description of the statistical parameters including central tendency (e.g. means) or other basic estimates (e.g. regression coefficient) AND variation (e.g. standard deviation) or associated estimates of uncertainty (e.g. confidence intervals)
☐	☒	For null hypothesis testing, the test statistic (e.g. F, t, r) with confidence intervals, effect sizes, degrees of freedom and P value noted<br>*Give P values as exact values whenever suitable.*
☒	☐	For Bayesian analysis, information on the choice of priors and Markov chain Monte Carlo settings
☒	☐	For hierarchical and complex designs, identification of the appropriate level for tests and full reporting of outcomes
☒	☐	Estimates of effect sizes (e.g. Cohen's d, Pearson's r), indicating how they were calculated

Our web collection on statistics for biologists contains articles on many of the points above.

Software and code

Policy information about availability of computer code

Data collection	Transmission electron microscopy (TEM) (HT7700, Hitachi, Japan);<br>Scanning electron microscopy (SEM) (S4800, Hitachi, Japan);<br>Universal material testing machine (3365, Instron, USA);<br>D8 Advance X-ray diffractometer (Bruck, Germany);<br>Nano-in Xider(Xenocs, France);<br>ABI Stepone Plus (Ambion, Foster City, CA, USA);<br>Fluorescence microscope (BX51, Olympus, Tokyo, Japan);<br>Chemiluminescence detection system (1708370, Bio-Rad, USA)
Data analysis	OMNIC 9 software (Thermo Scientific) ; PeakFit software (Seasolve, version 4.12)<br>Origin2018 software and OriginPro 2022b software; GraphPad Prism (v8.0.2, GraphPad)<br>FIT2D software; Image Lab (Bio-Rad, USA);GraphPad Prism (v8.0.2, GraphPad); Excel software (Microsoft365)

For manuscripts utilizing custom algorithms or software that are central to the research but not yet described in published literature, software must be made available to editors and reviewers. We strongly encourage code deposition in a community repository (e.g. GitHub). See the Nature Portfolio guidelines for submitting code & software for further information.

Data

Policy information about availability of data

All manuscripts must include a data availability statement. This statement should provide the following information, where applicable:

- Accession codes, unique identifiers, or web links for publicly available datasets
- A description of any restrictions on data availability
- For clinical datasets or third party data, please ensure that the statement adheres to our policy

NCBI silkworm genome database (SilkDB, https://www.ncbi.nlm.nih.gov/genome/?term=Bombyx+mori) was used in the study. All data generated in this study are available within the article, Supplementary Information and Source Data files. Source data are provided with this paper.

Field-specific reporting

Please select the one below that is the best fit for your research. If you are not sure, read the appropriate sections before making your selection.

☒ Life sciences ☐ Behavioural & social sciences ☐ Ecological, evolutionary & environmental sciences

For a reference copy of the document with all sections, see nature.com/documents/nr-reporting-summary-flat.pdf

Life sciences study design

All studies must disclose on these points even when the disclosure is negative.

Sample size	In the biocompatibility assay, three duplicate samples were set in each experimental group (Lin et al., 2021; Roca et al., 2020). In gene expression analysis and Western blotting, three duplicate samples were set in each experimental group. Each sample was taken from the tissues of three silkworm and mixed in equal quantities (Zhang et al., 2019; Li et al., 2015; Ji et al., 2013). In the silk fiber performance measurement, 20 individual silk fibers were tested in each experimental group (Peng et al., 2019).<br>1. Lin L, Zhou Y, Quan G, Pan X, Wu C. The rough inhalable ciprofloxacin hydrochloride microparticles based on silk fibroin for non-cystic fibrosis bronchiectasis therapy with good biocompatibility. Int J Pharm. 2021, 607:120974. doi: 10.1016/j.ijpharm.2021.120974.<br>2. Roca FG, Picazo PL, Pérez-Rigueiro J, Tortuero GVG, Pradas MM, Martínez-Ramos C. Conduits based on the combination of hyaluronic acid and silk fibroin: Characterization, in vitro studies and in vivo biocompatibility. Int J Biol Macromol. 2020, 148:378-390. doi: 10.1016/j.ijbiomac.2020.01.149.<br>3. Li Y, Chen X, Tang X, Zhang C, Wang L, Chen P, Pan M, Lu C. DNA synthesis during endomitosis is stimulated by insulin via the PI3K/Akt and TOR signaling pathways in the silk gland cells of Bombyx mori. Int J Mol Sci. 2015, 16(3): 6266–6280.<br>4. Zhang ZJ, Zhang SS, Niu BL, Ji DF, Liu XJ, Li MW, Bai H, Palli SR, Wang CZ, Tan AJ. A determining factor for insect feeding preference in the silkworm, Bombyx mori. PLoS Biol. 2019, 17(2):e3000162. doi: 10.1371/journal.pbio.3000162.<br>5. Ji MM, Liu AQ, Gan LP, Xing R, Wang H, Sima YH, Xu SQ. Functional analysis of 30K proteins during silk gland degeneration by a caspase-dependent pathway in Bombyx. Insect Mol Biol. 2013, 22(3):273–283。<br>6. Peng Z, Yang X, Liu C, Dong Z, Wang F, Wang X, Hu W, Zhang X, Zhao P, Xia Q. Structural and Mechanical Properties of Silk from Different Instars of Bombyx mori. Biomacromolecules. 2019, 20(3):1203-1216. doi: 10.1021/acs.biomac.8b01576.
Data exclusions	No data were excluded from the analyses.
Replication	To verify the reproducibility of the experimental finding, we performed biological replication and sample replication. In Western blotting ,qPCR and biocompatibility assay, 3 repeated samples were set in each experimental group, and each sample was equally mixed with 3 independent silkworm individuals. In the silk fiber performance measurement, n=22 cocoons in SER and n=27 cocoons in WT were tested. Image data are representative of three independent experiments unless otherwise stated. n=10 individual larva in silkworm growth and n=31 individual larva in cocoon silk production efficiency. Each experiment was repeated three times independently with similar results. all attempts at replication were successful.
Randomization	Silkworm individuals were randomly selected as samples except for gene expression analysis and Western blotting。 Samples for gene expression analysis and Western blotting were selected from male silkworm individuals in experiental groups.
Blinding	Blinding was not relevant to our study. Firstly, blinding is usually a research method that makes the experimenter and subjects unaware in human test. Moreover, we needed to identify and confirm the wild-type and mutants by fluorescence before all experiments in our study, which led to the inability to conduct blind experiments in our subsequent experiments.

Reporting for specific materials, systems and methods

We require information from authors about some types of materials, experimental systems and methods used in many studies. Here, indicate whether each material, system or method listed is relevant to your study. If you are not sure if a list item applies to your research, read the appropriate section before selecting a response.

Materials & experimental systems

n/a	Involved in the study
☐	☒ Antibodies
☐	☒ Eukaryotic cell lines
☒	☐ Palaeontology and archaeology
☐	☒ Animals and other organisms
☒	☐ Human research participants
☒	☐ Clinical data
☒	☐ Dual use research of concern

Methods

n/a	Involved in the study
☒	☐ ChIP-seq
☒	☐ Flow cytometry
☒	☐ MRI-based neuroimaging

Antibodies

Antibodies used

The primary antibodies used were: rabbit anti-SER3 and rabbit anti-P25 (synthesized by Wuhan GeneCreate Biological Engineering Co., Ltd.), mouse anti-EGFP antibody (ab184601,abcam,UK) and mouse anti-beta-Tubulin(ab108342,abcam,UK). All primary antibodies were diluted 1:2000.
The secondary antibodies used were: HRP-labeled goat anti-rabbit IgG and HRP-labeled goat anti-mouse IgG (Bioworld Technology, Minneapolis, MN, USA).All secondary antibodies were diluted 1:5000.
Except for rabbit anti-SER3 and rabbit anti-P25, the others are commercial antibodies.

Validation

All antibodies were validated by the company using western blots and Elisa.
First, the corresponding genes of the antigen fragment were selected, peT-B2M and PET-SUMo vectors were connected to transform the E. coli host, and the best expression strains were screened. One of them was selected to prepare the antigen and immunized Japanese white rabbits. The antibodies were purified by Protein A and verified by WB (anti-serum-recombinant antigen). And meet the following conditions:
1. Serum Elisa showed that the serum dilution ratio reached 1:32,000, and the OD value of 450nm was greater than 1 at 1:4000 dilution.
2. Antibody Elisa results show that the OD value of 450nm is greater than 1.0 when the concentration of coated antigen is 1.25ug/ mL and the concentration of detected antibody is 0.25ug/ mL.
3. The gene sequencing of the expression vector was correct (without any amino acid mutation or deletion);
4. Purity of antibody > 90% (SDS-PAGE);
5. The WB detection band of the provided antibody and recombinant antigen is correct.
6. The titer of recombinant antigen for antibody detection reached 1:50,000.

Eukaryotic cell lines

Policy information about cell lines

Cell line source(s)

RAW264.7 cell lines (ZQ0098, ZQXZ Biotech, Shanghai, China) and L-929 mouse fibroblasts cell lines (ZQ0093, ZQXZ Biotech, Shanghai, China) were used in this study.

Authentication

RAW 264.7 cells were derived from tumors induced by Murine leukemia virus Abelson. SIg -, Ia- antigen and THy-1.2 surface antigen were negative. RAW 264.7 cells did not secrete detectable virus particles and XC spot formation test was negative. RAW 264.7 cells could pinocytosis neutrophils and phagocytose latex particles and yeast glycan, and could decompose sheep red blood cells and tumor target cells in an antibody dependent manner. LPS or PPD treatment for 2 days induced red blood cell decomposition of RAW 264.7 cells, but had no effect on tumor target cells. The authentication procedures for L-929 mouse fibroblasts cell lines are the same as those for RAW264.7 cell lines.

Mycoplasma contamination

All cell lines were tested negative for mycoplasma.

Commonly misidentified lines
(See ICLAC register)

No.

Animals and other organisms

Policy information about studies involving animals; ARRIVE guidelines recommended for reporting animal research

Laboratory animals

The classic genetic strain N4W and the mutant SER of bombyx mori was used in this study. The laboratory animals included the fourth and fifth instar and pupal stages of silkworm. Unless otherwise stated, females individuals were used in the experiments.

Wild animals

No.

Field-collected samples

No.

Ethics oversight

No ethical approval or guidance was required. Because silkworm is a domesticated animal, it eats mulberry leaves or artificial feed, and will not escape. It can not reproduce in nature, nor is it limited by ecological concerns and ethical issues.

Note that full information on the approval of the study protocol must also be provided in the manuscript.
